# Place-of-residence errors on death certificates for two contiguous U. S. counties

**DOI:** 10.1186/1478-7954-4-6

**Published:** 2006-06-26

**Authors:** J Rush Pierce, Anne V Denison

**Affiliations:** 1 From the City of Amarillo Department of Public Health (Denison, Pierce); the Amarillo Bi-City-County Health District (Denison, Pierce), and the Division of Preventive Medicine, Department of Internal Medicine, Texas Tech University Health Sciences Center (Pierce), Amarillo, Texas

## Abstract

**Background:**

Based on death certificate data, the Texas Department of Health Bureau of Vital Statistics calculates age adjusted all-cause mortality rates for each Texas county yearly. In 1998 the calculated rates for two adjacent Texas counties was disparate. These counties contain one city (Amarillo) and are identical in size. This study examined the accuracy of recorded county of residence for deaths in the two counties in 1998. In our jurisdiction, the county of residence is assigned by funeral homes.

**Methods:**

A random sample of 20% of death certificates was selected. The accuracy of the county of residence was verified by using a large area map, Tax Appraisal District records, and U.S. Census Bureau databases. Inaccuracies in recording the county or zip code of residence was recorded.

**Results:**

Eighteen of 354 (5.4%) death certificates recorded the incorrect county and 21 of 354 (5.9%) of death certificates recorded the zip code improperly. There was a 14.4% county recording error rate for one county compared to a 0.82% for the other county. The zip code error rate was similar for the two counties (5.9% vs. 5.8%). Of the county errors, 83% occurred for addresses within a zip code that contained addresses in both counties.

**Conclusion:**

This study demonstrated a large error rate (14%) in recording county of residence for deaths in one county. A similar rate was not seen in an adjacent county. This led to significant miscalculation of mortality rates for two counties. We believe that errors may have arisen in part from use of internet programs by funeral homes to assign the county of residence. With some of these programs, the county is determined by zip code, and when a zip code straddles two counties, the program automatically assigns the county whose name appears first in the alphabet. This type of error could be avoided if funeral homes determined the county of residence from Tax Appraisal District or Census Bureau records, both of which are available on the internet. This type of error could also be avoided if vital statistics offices verified the county and zip code of residence using official sources.

## 

Death certificates represent a data source that many health researchers find useful and attractive for analysis. Along with birth certificates, they are the basis of many of the key indicators used for the comparison of health among populations. Though the amount of data contained in each death certificate is limited, essentially 100% of the deaths for a given period are represented. The accuracy of data contained on the death certificate has been the subject of a number of studies [1,2]. The major death certificate error that has been cited in the literature is inaccurate cause of death as assigned by certifying physicians [1-4]. Since death certificate data is used to calculate vital statistics, these inaccuracies lead to problems in reporting vital statistics and in population based studies that rely on vital statistics. This limitation has been recognized in the United States, Great Britain, Canada, and many other countries [1-15]. Several solutions for correcting these inaccuracies have been proposed, including physician education, quality assurance programs with feedback, and encouraging more autopsies [1,16-19]. All of these measures are designed to improve the accuracy of assigning the cause of death.

Data other than cause of death are also recorded on death certificates. In Texas, these include demographics (age, race, address, county of residence, and zip code of the deceased); date and time of death; if an autopsy was performed; if the deceased was pregnant at the time of death; whether or not tobacco and alcohol contributed to the death; if the death was natural or due to an accident, homicide, or suicide; and data related to occupation and educational level.

In most reports, the demographic data (place of death, residence of deceased, time of death, race/ethnicity) are assumed to be accurate and not subject to significant error. A study of the death certificate racial classification of American Indians in Montana found non-random biases associated with geographic location (proximity to a reservation), causes of death, and educational level [20]. Another study noted inaccuracies in recording the performance of autopsies [21]. A previous study found some errors in recording place of residence on death certificates, but these errors have tended to be uncommon [22].

We recently encountered a non-random demographic error in assigning county of residence on death certificates in two contiguous counties that resulted in significantly inaccurate mortality rates reported for these counties.

## Background

In many jurisdictions in Texas (including our city of Amarillo) death certificates are initiated by funeral homes. Clerical employees enter the basic demographic data (including education and occupation) and forward the death certificate to the attending physician, who completes the clinical data, including cause of death, time of death, whether or not tobacco or alcohol contributed to the death, and whether the death was natural or due to accident, homicide, or suicide. In Texas, death certificates are recorded by municipalities for deaths occurring within that municipality, and forwarded to the Texas Department of State Health Services Bureau of Vital Statistics.

The city of Amarillo straddles two Texas counties (Potter and Randall) that are contiguous and nearly equal in size and population. Using death certificate information provided by municipalities (cities and counties), the Texas Department of State Health Services Bureau of Vital Statistics calculates and publishes yearly, age-adjusted death rates, as well as disease specific death rates, for all counties in Texas.

As published by the Texas Department of Health (now the Texas Department of State Health Services), the 1998 age-adjusted death rates for these two contiguous counties (Potter and Randall) were 702 and 353 per 100,000. The same rate in all of Texas for 1998 was 513. These contiguous counties ranked one and 25 (of 33) respectively for age-adjusted deaths among all Texas counties with estimated 1998 populations greater than 100,000.

This marked disparity in age-adjusted deaths between the counties had been widening for several previous years (Table 1). Though there are demographic differences in poverty rates and ethnicity between the two counties (Table 2), the demographics had not changed significantly over the past decade. This disparity became the subject of much discussion at the local Health Department and in the community [23,24].

**Table 1:** Age adjusted death rates for Texas and for Potter and Randall counties, 1995 – 1998. Total yearly deaths included for Potter and Randall counties. Source: Texas Department of State Health Services, Texas Health Data [27]

Year	Potter County Rate/(Number)	Randall County Rate/(Number)	Texas Rate
1998	702 (1183)	354 (592)	513
1997	702 (1169)	400 (634)	516
1996	690 (1155)	405 (634)	519
1995	698 (1150)	431 (639)	526

**Table 2:** Selected Demographics for Potter and Randall Counties, Texas (source: Texas Department of Health, 1998 statistics)

	Randall County	Potter County
Population over age 64	11.6%	11.6%
Total Estimated 1998 Population	105,736	106,046
Anglo	89.0%	64.1%
Hispanic	8.3%	22.8%
Black	1.4%	9.2%
Other	1.3%	3.9%
Unemployment rate	1.6%	5.6%
Licensed Nursing Home Beds	296	1,285

In 2000, the City of Amarillo Department of Public Health designed and conducted a study to verify the accuracy of the county of residence as entered on 1998 death certificates for residents living within the Amarillo city limits. The two major questions asked were (a) do errors on the death certificate have a significant effect on county mortality data, and (b) what is the most likely source of these errors. This study was exempted from review by the Institutional Review Boards of the Texas Department of Health and the Texas Tech University Health Sciences Center.

## Methods

After signing notarized confidentiality statements, two Health Department nurses reviewed every seventh death certificate for 1998 deaths in Amarillo (records were filed alphabetically). If the seventh death certificate was for a citizen who resided outside of Amarillo, the previous death certificate was reviewed. Street address, zip code, and county of residence at the time of death; and the agency that filed the death certificate was determined for each selected death certificate and entered into an ACCESS database. Names were not recorded in order to protect confidentiality.

For each selected death certificate, correct zip code and county of residence were determined using a large-scale map (Revision date, January 2000) obtained from the City of Amarillo Planning Department. If the county of residence or zip code could not be accurately determined using the map, a query was made to the county Tax Assessors office or the information was obtained from the Potter-Randall Tax Appraisal District website [25]. Correct county and zip code of residence for all suspected errors were later confirmed using the Census Bureau website [26]. A "county error" was defined as a residence recorded as Potter County on the death certificate which was actually located in Randall County or a residence listed in Randall County, which was actually located in Potter County. "Zip code error" was defined as an incorrectly recorded zip code. Routine statistical tests were used to analyze error rates by correct county of residence, correct zip code of residence, and agency filing the death certificate.

## Results

A total of 362 death certificates were selected for review. Eight death certificates were discarded due to unverifiable addresses (street did not exist in Amarillo or Post Office Box addresses), leaving a total of 354 death certificates in the study. This represents 98% of the selected death certificates, and 20% of the 1,775 deaths assigned to Potter and Randall counties for 1998 based on vital statistics available from the Texas Department of Health. Of the 354 death certificates reviewed, 242 (69%) were in Potter County and 31% were in Randall County. This is consistent with the total death statistics in 1998 as reported by the Texas Department of Health (1,183 of the 1,775 deaths in the two county area, or 66%, occurred in Potter County).

Eighteen errors in assigning the proper county of residence ("county errors") were detected. The overall error rate for county-of-residence data entered on the death certificates reviewed was 5.4% (18/354). Twenty-one "zip code errors" were identified, for an error rate of 5.9%. In general, errors in county and errors in zip code occurred independently of one another. Two death certificates contained inaccuracies in both county and zip code.

The percentage of error was markedly different for the two counties (Table 3). There was a 14.4% error rate for Randall County (i.e. addresses for residence in Randall County recorded as in Potter County) as compared to an error rate of 0.82% for Potter County. This non-random pattern of error was statistically significant (Pearson Chi Square, p < 0.001). This pattern of non-random error was not observed in the zip code data where the overall error rate was 5.9% with a rate of 5.8% for Potter County and 6.3% for Randall County.

**Table 3:** Death certificate errors. Number of errors in assigning county and zip code for City of Amarillo residents, 1998.

County of residence	Records reviewed	County errors (%)	Zip code errors (%)
Potter	243	2 (0.82)	14 (5.8)
Randall	111	16 (14.4)	7 (6.3)

Incorrect county errors were confined to addresses in only five of 13 zip codes (Table 4). Eighty-three percent (15/18) of the errors occurred for addresses in zip codes that contain addresses in both counties (shared zip codes). Forty-eight percent (48%) of Potter County deaths occurred in residents living in shared zip codes compared to 27% of Randall County deaths.

**Table 4:** County errors by zip code of residence. Number of county errors by zip code of deceased, and counties represented within each zip code (P = zip code area includes Potter county addresses only; R = zip code area includes Randall county addresses only; PR = zip code area includes addresses in both counties)

Zip code	County code	Records reviewed	Errors	Rate
79101	P	12	0	
79102	P	20	0	
79104	P	11	0	
79107	P	68	0	
79108	P	15	0	
79110	R	29	3	
79119	R	1	0	
Total for one county zipcodes		156	3	1.9%
79103	PR	20	2	
79106	PR	83	5	
79109	PR	75	7	
79118	PR	6	0	
79121	PR	8	1	
79124	PR	6	0	
Total for two county zipcodes		198	15	7.6%
Total for all zipcodes		354	18	5.1%

Errors were analyzed by the funeral home that filed the death certificate. Funeral homes from outside of the two county area accounted for more county errors than funeral homes within the two county area (Pearson Chi Square, p = 0.04). Outside funeral homes accounted for 7.9% of the total death certificates reviewed but were responsible for 39% of the county errors and 29% of the zip code errors.

Correcting the mortality figures for the error rate found in this study would result in approximately 65 fewer deaths assigned to Potter County in 1998, would lower the 1998 age adjusted all-cause mortality rate for Potter county and raise the 1998 age adjusted all cause mortality rate for Randall county. Using recalculated crude mortality rates, the rankings of these counties would change from first to third (Potter) and 25th to 20th (Randall) in the list of 33 counties with populations over 100,000.

## Comment

This study found that in a large sample, over 14% of death certificates for residents of Randall County were incorrectly recorded as residing in Potter County, whereas less than 1% of Potter county residents were incorrectly recorded as living in Randall County. An error in recording the incorrect county was much more likely when persons lived in a zip code that straddled both counties, and when the death certificate was completed by an out-of-town funeral home compared to a local funeral home.

We believe that this error rate may have arisen in part from use of internet programs by funeral homes to assign the county of residence. With some of these programs, the county is determined by zip code, and when a zip code straddles two counties, the program automatically assigns the county whose name appears first in the alphabet. An informal survey of funeral homes conducted by the Department verified that funeral homes often used these types of internet programs when the family or funeral home was uncertain of the county of residence. This scenario would explain why county errors were more likely in zip codes areas that straddled two counties, and why the incorrect assignment was more likely to be to Potter than Randall County, as P precedes R in the alphabet. Numerically, a larger proportion of Potter County deaths occurred in these shared zip codes (45% vs. 28% for Randall County). Since more county-of-residence errors occurred in these shared zip codes, this offers another explanation why county-of-residence errors were higher in Potter County than in Randall County.

Amarillo is an unusual Texas city that straddles two counties. Thus compared to citizens in other cities, Amarillo citizens may be less certain of their county of residence. Therefore this observation may not be generalizable to all Texas cities.

This type of error led to significant miscalculation of the age-adjusted all cause mortality for these two counties, as well as incorrect calculation of age-adjusted disease specific death rates for these counties. While previous reports have noted inaccuracies in disease specific mortality rates that are derived from death certificates, we believe that ours is the first report of inaccurate all-cause mortality rates for contiguous counties due to a nonrandom error in assigning the county of residence. We believe that this type of error could be avoided by verification of the county of residence by using tax appraisal district records or census bureau records, both of which are available on the internet. This type of error could also be avoided if vital statistics offices verified the county and zip code of residence using official sources, or used geocoding programs such as TIGER. This program is used in some states to assign city, county, and zip code based on the street address reported on the death certificate. We report this study to remind demographers of additional limitations of vital statistics derived from death certificate information as it is now collected.

**Table 5:** Errors by funeral home location. Local means that the funeral home was located within Potter or Randall county.

Location of funeral home	Death certificates reviewed	County errors (rate)	Zip code errors (rate)
Local	326	11 (3.4%)	15 (4.6%)
Not local	28	7 (25%)	6 (21%)
